# Therapeutic Strategies for Metastatic Triple-Negative Breast Cancers: From Negative to Positive

**DOI:** 10.3390/ph14050455

**Published:** 2021-05-12

**Authors:** Dey Nandini, Aske Jennifer, De Pradip

**Affiliations:** Translational Oncology Laboratory, Avera Cancer Institute, Sioux Falls, SD 57105, USA; Nandini.Dey@avera.org (D.N.); Jennifer.Aske@avera.org (A.J.)

**Keywords:** triple-negative breast cancer (TNBC), targeted therapy, immunotherapy

## Abstract

Metastatic triple-negative breast cancer (TNBC) is a distinct and immensely complex form of breast cancer. Among all subtypes of breast cancers, TNBC has a comparatively high rate of relapse, a high rate of distant metastasis, and poor overall survival after standard chemotherapy. Chemotherapy regimens are an essential component of the management of this estrogen receptor-negative, progesterone receptor-negative, and epidermal growth factor receptor2 negative subtype of breast cancers. Chemotherapy is critical for preventing the recurrence of the disease and for achieving long-term survival. Currently, a couple of agents are approved for the management of this disease, including chemotherapy like eribulin, targeted therapy like PARP inhibitor, as well as an antibody-drug conjugate (ADC) to target TROP2. Like many other metastatic cancers, immune checkpoint inhibitors (ICIs) have also been approved for TNBC patients with PD-L1 positive tumors and high tumor mutational burden. In this review article, we discuss these newly approved and promising novel agents that may change the therapeutic landscape for advanced/metastatic TNBC patients.

## 1. Background

Triple-negative breast cancer is conventionally/immunohistochemically defined by the lack of estrogen receptor (ER), progesterone receptor (PR), and epidermal growth factor receptor 2 (HER2) expression. Clinically this subtype of breast cancer is more sensitive to chemotherapy than other subtypes of breast cancers (such as ER+ and *HER2* amplified). However, it is also characterized to harbor the most aggressive behavior with the risk of relapse within 3 to 5 years after completion of adjuvant chemotherapy and also its commonness in younger women as well as more prevalent in African American women [1,2,3,4]. The incidence of TNBC is disproportionately higher in African American (AA) women, for which there are several reasons. Socioeconomic factors may contribute to more impoverished survival conditions (low income, low education, less healthy lifestyle, and low access to the health care system). Preclinical and clinical studies show that inherent genetic aberrations of *TP53* , *BRCA1*, *AURKA*, *AURKB*, * PLK1*, and *EZH2* occur in this population at a disproportionately higher rate [5,6]. The high Incidence of obesity influences various signaling pathways related to aggressive tumor progression, including growth and metastasis [7]. Regimens that include three classes of chemotherapy drugs, namely anthracyclines, cyclophosphamide (DNA alkylator/crosslinker), and taxanes, represent the global standard of care for the patient with TNBC [8]. Recent data from the SYSUC-001 randomized clinical trial also revealed that women with early-stage TNBC who received standard adjuvant treatment—a low maintenance dose of capecitabine therapy for 1 year compared with observation—resulted in significantly improved disease-free survival for 5 years (85.8 vs. 75.8%; HR: 0.60) [9]. In November 2010, the FDA approved eribulin in patients with metastatic BC following administration of at least two regimens with an anthracycline and a taxane, based on data from the EMBRACE trial, which showed statistically significant development in overall survival for patients treated with eribulin compared to those treated with therapy of the physician’s choice [10]. Eribulin is a non-taxane synthetic analog of halichondrin B, which inhibits microtubule polymerization without affecting depolymerization, thus causing less toxicity compared to taxanes. In this study, 19% of all cases had a TNBC, and eribulin was greatly effective, with its use being associated with a 29% reduction in risk of death compared to other treatments. Although the mainstay of treatment of metastatic TNBC patients is chemotherapy, novel genomically-driven targeted agents and immunotherapy agents have been successfully incorporated in the routine clinical practice or late stage in the clinical trial. Frequent genetic alterations found in DNA damage pathways including germline as well as somatic *BRCA1/2* genes, upregulation of the PI3K-AKT pathway molecules, the presence of androgen receptors (AR), the trophoblast cell-surface antigen (TROP2), and infiltrating T-cells as well its cognate ligand (PD-L1/L2) might serve as actionable targets to optimize the treatment options in the era of precision medicine and improve the outcome of patients with TNBC. There is an unmet need to improve clinical outcomes when treating TNBC, particularly in the context of patients with advanced/metastatic disease.

## 2. Molecular Subtypes of TNBC

**Figure 1:**
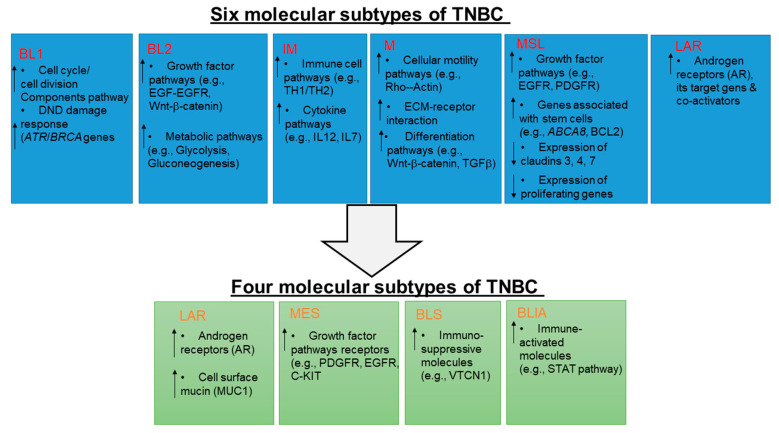
Schematic illustration of the molecular subtypes of triple-negative breast cancers. The blue and green boxes are six subtypes and four different subtypes of triple-negative breast cancer (TNBC), respectively, with their characteristic features. The six subtypes are **BL1**, basal-like 1; **BL2**, basal-like 2; **IM**, immunomodulatory; **M**, mesenchymal; **MSL**, mesenchymal stem-like; **LAR**, luminal androgen receptor. Four subtypes are **LAR**, luminal AR; **MES**, mesenchymal enrich; **BLIS**, basal-like immunosuppressive; **BLIA**, basal-like immune activated.

According to clinical, pathological, and genetic factors, emerging evidence indicates that TNBC is a molecularly diverse disease with an unpredictable prognosis. The molecular stratification of TNBC patients has been critical in identifying targeted therapeutic opportunities in the era of precision medicine. A decade ago, Lehmann and colleagues identified six TNBC subtypes based on the unique gene expression profiling. Although, each of these subtypes has different as well as overlapping clinical-pathological and molecular risk features. The subtypes are (1) basal-like (**BL1**; heavily enriched with cell cycle/cell division components pathway, DNA damage response (ATR/BRCA) pathways genes), (2) basal-like (**BL2;** enriched with growth factor signaling pathways, Wnt/β-catenin, metabolic pathway-related genes), (3) an immunomodulatory (**IM;** enriched with immune cell signaling and cytokines signaling pathways genes), (4) a mesenchymal (**M;** enriched in components and pathways involved in cell motility, ECM receptor interaction, and cell differentiation pathway-related genes), (5) a mesenchymal stem-like (**MSL;** genes representing components and processes linked to growth factor signaling pathways that include inositol phosphate metabolism, EGFR, PDGF, calcium signaling, G-protein coupled receptors, and ERK1/2 signaling as well as ABC transporter and adipocytokine signaling pathway genes), and (6) luminal androgen receptor (**LAR;** highly enriched androgen receptor and its downstream androgen targets and co-activators genes) subtype [11]. Later, Burstein and colleagues simplified TNBC tumors into four different subtypes via RNA and DNA profiling analysis. Those subtypes are (a) luminal AR (**LAR**; enriched in androgen receptors, cell surface mucin, MUC1, FOXA1, amplification of CCND1), (b) mesenchymal (**MES;** enriched in growth factor receptors like PDGFR1, EGFR, c-KIT), (c) basal-like immunosuppressed (**BLIS;** enriched in immunosuppressive molecules like VTCN1, amplification of FGFR2), and (d) basal-like immune-activated (**BLIA**, STAT signaling molecules, cytokines, amplification of CDK1) (Figure 1). The subclassification was utilized to stratify prognosis. BLIS and BLIA tumors have the worst and best prognosis, respectively [12].

## 3. Current FDA Approved Treatment Options

### 3.1. Alterations of DNA Damage Signaling Pathway and Targeted Therapeutics

As described before, TNBC includes molecularly different subgroups. One of them harbors defects in DNA repair (homologous recombination, HR) genes. Out of several genes, *BRCA1/2* alterations (copy number loss, mutations, or methylation) are well-established drug targets to control tumorigenesis, as well as management of the disease, including TNBC patients. *BRCA* mutations compromise the ability of the tumors to recover from the DNA damaging agents by reducing their capacity to DNA repair by HR [13,14]. It is well established that BRCA1/2 signaling plays a critical role in higher reliability of DNA repair at the point of DNA double-strand break via the processes of HR and also through the activation of other DNA repair pathway genes (e.g., *RAD51*, *BAP1*) [15]. TNBC patients with *BRCA1/2* mutations lack effective DNA repair activity. However, it is uncertain whether somatic *BRCA* alterations or promoter methylation leads to exactly demonstrate the same function deficiency as germline *BRCA1/2* mutations.

Ten percent of TNBC patients harbor *BRCA1/2* copy number loss or loss-of-function mutations. Platinum-based chemotherapy regimens have engrossed some attention as a potential therapeutic opportunity for TNBC patients, and their clinical use has been supported by a realistic association of TNBC tumors with germline mutations of *BRCA1/2* genes and [3,16]. Unfortunately, platinum-based chemotherapies have shown limited benefit in general metastatic breast cancer patients. Some randomized trials addressed the efficacy of platinum-based chemotherapy regimens in metastatic TNBC. *BRCA1/2* loss-of-function mutated tumors exhibited defective HR and demonstrated synthetic lethality with poly (ADP ribose) polymerase (PARP) inhibitor in different solid tumors, including TNBC [17,18]. TNBC tumors harboring defeats in DNA-damage repair genes like *BRAC1/2* respond to inhibitors of the DNA-damage repair pathway as they rely on the intact components of the repair pathway like PARP.

**Figure 2:**
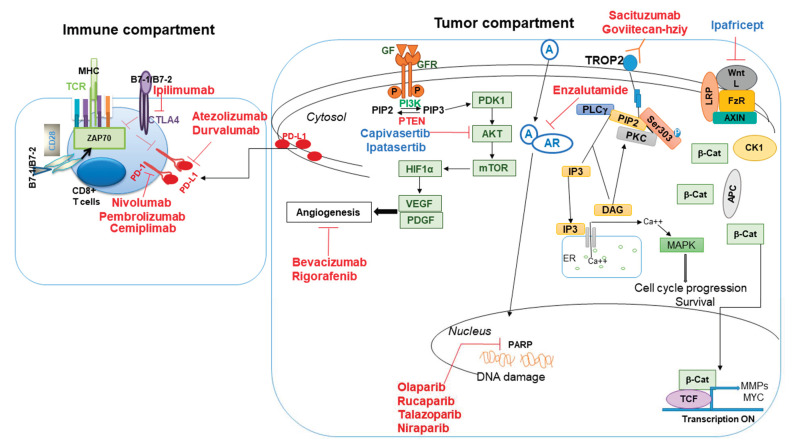
Targeted and immunotherapies in TNBC clinical studies. Several targeted drugs in the TNBC tumor and immune compartments have been explored either with a single agent or combinations to attack the tumor-microenvironment. Various signaling targets (including the PI3K-AKT-mTOR pathway; Wnt-beta-catenin pathway), DNA damage response pathway (targeting PARP), critical/relevant immune checkpoint pathway (PD-L1-PD-1 axis, CTLA4), angiogenic pathway (HIF1 alpha-VEGF), and ADC target (TROP2-mediated ADC) are shown. The molecular landscape of TNBC (including immune compartment) confers insight into the novel and investigational targeted therapies, which are directly confronting its heterogeneous biology, cellular signaling pathways, and their importance for targeting in TNBC tumor. This molecular landscape provides insight into the heterogeneous biology and rationale for targeted therapies. AR, androgen receptor; RED, FDA approved drugs; BLUE, FDA non-approved drugs but active in clinical trials.

The family of PARP enzymes is a DNA-damage sensor-mechanism in cells that use NAD+ and is most active in the S-phase of the cell cycle, in its critical roles in DNA repair [19]. Small molecule NAD+ mimetics like olaparib, talazoparib, niraparib, rucaparib, and veleparib inhibit the catalytic activity of PARP (Figure 2). PARP inhibitors have been known to produce robust and dependable clinical benefits by virtue of “synthetic lethality.” Olaparib and talazoparib are FDA approved for TNBC patients with germline BRCA1/2 alterations; others are currently being studied in the late stage of clinical trials as a single agent or a combination therapy.

A decade ago, Fong and colleagues published their clinical trial (phase 1) data of olaparib in patients with germline *BRCA1/2* mutation in breast and ovarian cancers. Their data demonstrated that one of nine breast cancer patients with a germline *BRCA2* mutation had a complete response, and an additional three out of nine patients, one with a germline *BRCA2* mutation and the other two with wild-type *BRCA*, had stable disease [20]. Olaparib is the first treatment approved in 2018 specifically for germline *BRCA* mutation carriers with HER2-negative metastatic breast cancer with previous treatment with chemotherapy in the neoadjuvant, adjuvant, or metastatic setting [21]. This approval was based on the high efficacy data of the OlympiAD trial. The phase III OlympiAD was an international, open-label, randomized trial that evaluated the efficacy and safety of olaparib in patients with metastatic HER2-negative and either estrogen receptor (ER)/progesterone receptor (PR)–positive or –negative breast cancer. Patients were required to have a known or suspected germline *BRCA1/2* mutation and received no more than two previous chemotherapy regimens. A total of 302 patients were randomized in a 2:1 fashion to olaparib at 300 mg twice daily or single-agent chemotherapy of the provider’s choice, including eribulin, capecitabine, or vinorelbine. This study demonstrated a higher median progression-free survival (PFS) in the olaparib group compared to the standard therapy group (7.0 months vs. 4.2 months, HR: 0.58, *p* < 0.001). The objective response rate (ORR) was doubled in the olaparib group compared to the standard therapy group (59.9 vs. 28.8%). The second progression was also observed to be longer in the olaparib arm (HR: 0.57). Olaparib monotherapy provided the statistically significant and clinically meaningful benefit of PFS to HER2 negative metastatic breast cancers with a germline mutation of *BRCA1/2* [22].

In the later part of 2018, the FDA also approved talazoparib, an oral PARP inhibitor, to treat adults with deleterious or suspected deleterious germline *BRCA* mutation-positive *HER2*-negative locally advanced or metastatic breast. [23]. It is required that the presence of a germline *BRCA* mutation must be identified by the FDA-approved companion diagnostic *BRAC* Analysis CDx test. Turner and his group reported from their phase II ABRAZO trial data, where they investigated talazoparib in patients with germline *BRCA1/2* mutations in locally advanced or metastatic TNBC, with or without prior exposure to a platinum-containing agent. Response rates to talazoparib were significantly poorer in patients who had received prior platinum-containing therapy. The objective response rate (ORR) and clinical benefit rate (CBR) were 20.8% and 27.1%, respectively, in the platinum-exposed patients. For the patients without platinum exposure, ORR was 37.1%, and CBR was 45.7% [24]. Similar to olaparib, talazoparib was also approved due to the high efficacy data of EMBRACA trail. The phase III EMBRACA trial compared talazoparib with physician’s choice chemotherapy (eribulin, vinorelbine, capecitabine, or gemcitabine) in patients with advanced breast cancer and germline *BRCA1/2* mutations. The median mPFS was 8.6 months in the talazoparib arm compared to 5.6 months in the chemotherapy arm (HR: 0.54, *p* < 0.0001) with an ORR of 62.6% with talazoparib compared to 27.2% with chemotherapy [25]. Single-agent talazaparib significantly prolonged PFS in HER2 negative advanced breast cancers with a germline *BRCA1/2* mutation as compared to the patients treated with chemotherapy of physician’s choice. Furthermore, all secondary efficacy end-points (DOR, OS) demonstrated benefit with talazoparib. Both the PARP inhibitors are well managed in clinics; however, toxicity includes gastrointestinal side effects, fatigue, and myelosuppression.

Recently Chopra et al. reported on the activity of rucaparib in the TNBC subgroup harboring evidence of defective HR DNA repair status. Within the trial group, investigators have recruited germline *BRCA1/2* patients as a control population. They prospectively examined the three potential biomarkers of rucaparib activity, a molecular signature of HR deficiency, RAD51 focus formation in tumor biopsy at the end of the treatment, and BRCA1 methylation. They also assessed rucaparib activity by Ki67 as well as cleaved PARP status. Rucaparib activity was also assessed by circulating tumor DNA (ctDNA) dynamics. No association was observed between Ki67 with *BRCA1/2* mutation as well as cleaved PARP level with *BRCA1/2* mutated cancer patients. However, the level of ctDNA was significantly reduced in germline *BRCA1/2* mutated patients when compared with *BRCA1/2* wild-type counterpart. More importantly, their study also demonstrated that rucaparib induces pro-inflammatory/interferon response in HR deficient TNBC, likely through the cGAS–cGAMP–STING pathway. Data indicated that PARP inhibitors might combine with PD-1/PD-L1antibodies for better outcomes of germline *BRCA1/2* mutated TNBC patients [26].

The initial phase I study with niraparib (NCT00749502) showed a partial response with RECIST criteria with a small number of *BRCA1/2* germline mutated breast cancer patients [27]. A phase III, randomized, open-label, multicenter, controlled trial of niraparib vs. physician’s choice in previously-treated, HER2-negative, germline *BRCA* mutation-positive breast cancer patients has been opened (An EORTC-BIG intergroup study (BRAVO study), NCT01905592). Niraparib is more active with immune checkpoint inhibitors in TNBC patients. BROCADE3 study (NCT02163694) showed that the addition of veliparib to a highly active platinum doublet, with continuation as monotherapy if the doublet were discontinued, resulted in significant and durable improvement in median PFS [14.5 months in the veliparib group vs. 12.6 months in the control group (HR: 0.71)] in patients with germline *BRCA* mutation-associated advanced breast cancer [28].

### 3.2. Immune Therapies in TNBC Patients

Among the breast cancer patients, the TNBC subsets are more immunogenic with a higher number of tumor-infiltrating lymphocytes (TILS) in their tumor-microenvironment, but it also showed a high level of PD-L1 expression [29,30] (see Figure 2). Hence, immune-modulation/immunotherapy (either PD-1 or PD-L1 antibody) is a good choice for the patient with PD-L1-positive status. Atezolizumab (anti-PD-L1 antibody), the first immunotherapy drug, was approved by FDA along with nab-paclitaxel in PD-L1+ (the threshold is ≥ 1%) advanced or metastatic TNBC patients following the IMpassion130 trial. FDA also approved the VENTANA PD-L1 (SP142) assay (PD-L1 positive tumor cells + tumor-associated immune cells) as a companion diagnostic device for selecting TNBC patients for atezolizumab. In patients whose tumors express PD-L1, median PFS was 7.4 months for patients receiving atezolizumab with nab-paclitaxel and 4.8 months for those receiving placebo with nab-paclitaxel (HR:0.60). In the first interim analysis, the median overall survival (OS) was 25 vs. 15.5 months among patients with PD-L1+ tumors [31]. This combination was well tolerated, although immune-related adverse events including rash, hypothyroidism, and pneumonitis were reported in patients [32].

In the later part of 2020, the FDA granted accelerated approval to pembrolizumab (anti-PD-1 antibody), in combination with chemotherapy, for the treatment of patients with locally recurrent unresectable or metastatic TNBC whose tumors express PD-L1 (CPS ≥ 10; combined positive score, which is the number of PD-L1 staining cells (tumor cells, lymphocytes, macrophages) divided by the total number of viable tumor cells, multiplied by 100). The FDA also approved the PD-L1 IHC 22C3 pharmDx (Dako North America, Inc.) as a companion diagnostic for selecting patients with TNBC for the treatment of pembrolizumab. Approval was based on KEYNOTE-355, a multicenter, double-blind, randomized, placebo-controlled trial in patients with TNBC, who had not been previously treated with chemotherapy (protein-bound paclitaxel, or paclitaxel, or gemcitabine plus carboplatin) in the metastatic setting. Median PFS was 9.7 months in the pembrolizumab plus chemotherapy arm and 5.6 months in the placebo arm (HR: 0.65). Overall the combination is well tolerated.

As mentioned previously, a cross-talk exists between PARP inhibition and the PD-1/PD-L1 axis. Preclinical models have demonstrated synergistic antitumor efficacy with the combination of PARP inhibitor and checkpoint inhibitor irrespective of *BRCA* mutation status as well as PD-L1 expression [33,34]. Several clinical studies also reported the mechanistic relationship of PARP inhibitor-induced PD-L1 expression via the activation of the cGAS–cGAMP–STING pathway [26,34].

They recently reported MEDIOLA phase I/II basket trial (NCT02734004) of durvalumab (anti-PD-L1 antibody) and olaparib in solid tumors, including germline *BRCA1/2*-mutated TNBC patients; the ORR was 53% and a 12-weeks DCR (disease control rate) of 47% [35]. Similarly, a combination of niraprib plus pembrolizumab (TOPACIO study) provided promising antitumor activity with a tolerable safety profile in patients with advanced or metastatic TNBC with numerically higher response rates in those with tumor *BRCA* mutations (PFS 8.3 months for tumor *BRCA* mutated patients vs. 2.1 months for *BRCA* wild type tumors) [36].

### 3.3. Antibody–Drug Conjugates (ADC) as a Targeted Therapy

Antibody-drug conjugates (ADCs) represent a promising therapeutic modality for the clinical management of cancer. ADCs are composed of recombinant chimeric, humanized, or human antibodies covalently bound by synthetic cleavable linkers to highly cytotoxic drugs. The primary objective is to combine the pharmacological potency of small (300 to 1000 Dalton) cytotoxic drugs with the high specificity of monoclonal antibodies that target tumor-associated antigens [37]. ADCs offer significant advantages over conventional chemotherapy. ADCs attach specifically to tumor cells with their target receptors while not affecting healthy cells as normal cells do not have sufficient receptors in question. Sacituzumab govitecan-hziy is an anti-trophoblast cell-surface antigen (TROP-2) antibody conjugated with a potent DNA damaging agent, an active metabolite of irinotecan (SN-38), by a pH-sensitive cleavable linker (Figure 2). TROP-2 is expressed in more than 90% of TNBCs [38], and its expression is associated with prognosis [39]. In 2020 FDA granted accelerated approval to sacituzumab govitecan-hziy for patients with metastatic TNBC who received at least two prior therapies for metastatic disease. Efficacy was demonstrated in IMMU-132-01 (NCT01631552), a multicenter, single-arm trial enrolling 108 patients with metastatic TNBC patients who had received a median of 3 previous therapies. The response rate (3 complete and 33 partial responses) was 33.3%, and the median duration of response was 7.7 months as assessed by independent central review; these values were 34.3% and 9.1 months, respectively. The clinical benefit rate was 45.4%, and median PFS was 5.5 months, and OS was 13.0 months [40]. Moreover, the combination of sacituzumab govitecan-hziy plus rucaparib is highly effective against TNBC patients, according to the results of the phase IB SEASTAR study presented at the 2020 virtual ESMO meeting by Professor of Timothy Yap from MD Anderson Cancer Center, Texas.

## 4. Promising Treatment Options with Drugs That Need FDA Approval

### 4.1. Antibody–Drug Conjugates (ADC)

Ladiratuzumab vedotin (LV) is a humanized antibody targeting the zinc transporter LIV-1 conjugated with a potent microtubule-disrupting agent, monomethyl auristatin E (MMAE), with a protease cleavable linker. LIV-1 is highly expressed in metastatic TNBC. LV monotherapy has demonstrated encouraging antitumor activity and tolerability in LIV-1 positive advanced or metastatic TNBC (NCT01969643). Among the 44 patients with TNBC in the combined dose-escalation and expansion cohorts, the ORR was 32%, and the median PFS was 11.3 weeks. [41]. Likewise, a phase IB/2 study (SGNLVA-002) evaluated safety, tolerability, activity, and recommended phase II dose of LV plus pembrolizumab (NCT03310957) in a dose-finding followed by expansion phase as front line therapy for TNBC patients. In this ongoing trial, 26 patients (unresectable locally advanced or metastatic TNBC patients) were followed for at least 3 months with an opportunity for two post-baseline disease assessments. Among these patients, the confirmed ORR was 54% [42]. NBE-002, an anthracycline-based immune-stimulatory humanized antibody-drug conjugate (iADC) targeting against the receptor tyrosine kinase ROR1, has been tested for the treatment of TNBC. ROR1 is expressed on the surface of numerous solid tumors, including TNBC. NBE-002 is site-specifically conjugated to a derivative of the highly potent anthracycline PNU-159682. The preclinical PDX model showed that NBE-002 is a highly effective and promising targeted therapeutics for the treatment of ROR1 positive TNBC [43]. ROR1 is expressed during embryo–fetal development but disappears before birth and is usually not expressed in normal cells in children or adults. However, ROR1 may reappear on malignant tissues and is also expressed across a wide variety of cancer types, including TNBC. The first in-human study will evaluate the recommended dose for further clinical development, safety, tolerability, antitumor efficacy, immunogenicity, pharmacokinetics, and pharmacodynamics of NBE-002 in patients with advanced solid tumors, including TNBC (NCT04441099) (Table 1).

Trastuzumab deruxtecan is a HER2 targeted ADC, and it is FDA approved for HER2+ metastatic patients and delivers a potent topoisomerase I inhibitor payload, which is linked to a humanized anti-HER2 antibody. Trastuzumab deruxtecan is the first HER2-targeted agent to demonstrate the promising clinical antitumor activity with a manageable safety profile in patients considered HER2-negative. Based on this, the DESTINY-Breast04 phase III trial (NCT03734029) was initiated to compare the efficacy and safety of Trastuzumab deruxtecan to physician’s choice chemotherapy (capecitabine, eribulin, gemcitabine, paclitaxel, or nab-paclitaxel) in patients with HER2-low, unresectable, and/or metastatic breast cancers [44].

### 4.2. Signaling Pathway-Targeted Therapies

PI3K-PTEN-AKT pathway: NGS-based molecular targeted therapy is the most important therapeutic opportunity in the era of precision medicine. There is a strong genomic and proteomic signature of the active PI3K-AKT pathway in TNBC subtypes, and this represents the major druggable signaling pathway (Figure 2). Unlike ER+ luminal breast cancers, where *PIK3CA* mutations (both at helical and kinase domain) dominate, the PI3K-AKT pathway activating events in TNBC include a broader spectrum of genes with a relatively lower frequency of *PIK3CA* mutations (9%) and much more frequent deletion/mutations/loss of negative regulators of PI3K, namely *PTEN* (35%) and *INPP4B* (30%) [45]. Additional gene amplification observed in this pathway includes *PIK3CA*, *AKT1*, *AKT2*, or *AKT3* [46]. All these alterations lead to a higher degree of AKT activation. Several novel molecularly targeted agents against the PI3K-AKT pathway have now been developed, including the AKT inhibitors capivasertib (AZD5363; AstraZeneca) and ipatasertib (GDC-0068; Genentech) are active in the later stage of clinical trials either alone or with combinations. Results from two placebo-controlled randomized phase II trials (LOTUS and PAKT) in metastatic TNBC provide encouraging evidence that AKT is a clinically relevant target in TNBC. A multicentre, randomized, double-blind, placebo-controlled, phase II study (LOTUS trial) with metastatic PI3K-AKT pathway activated TNBC patients demonstrated that ipataserib plus paclitaxel was significantly more efficacious than placebo control patients. Median PFS in the intention-to-treat population was 6.2 months with ipatasertib versus 4.9 months with placebo (HR: 0.60). In the 48 patients with PTEN-low tumors, median PFS was 6.2 months with ipatasertib versus 3.7 months with placebo (HR: 0.59) group. [47]. In the 2020 ESMO breast cancer virtual meeting, Dent and colleagues presented the LOTUS trial’s latest data. The median OS favored the ipatasertib combination across all biomarker-defined subgroups. Among patients with normal tumor IHC PTEN status, the median OS was 28.5 vs. 17.1 months with ipatasertib vs. placebo, respectively (HR: 0.70); and the 1-year OS rates were 85% versus 68%, respectively. Among patients with low PTEN status, the median OS and 1 year OS rates also favored the ipatasertib arm at 23.1 versus 15.8 months (HR: 0.83) and 79 vs. 64%, respectively. Interestingly, they also highlighted that among patients aged <50 years, the median OS was 35.2 months with ipatasertib vs. with 15.1 months with placebo (HR: 0.41). In patients aged ≥50 years, the median OS was 21.8 months with ipatasertib vs. with 20.9 months with placebo (HR: 1.21) [48].

Similar to the LOTUS study, the PAKT trial [49] also demonstrated encouraging data with capivasertib plus paclitaxel, especially in *PIK3CA*/*AKT1*/*PTEN*-altered metastatic TNBC patients. Capivasertib plus paclitaxel showed improved median PFS (5.9 months vs. 4.2 months; HR: 0.74) compared to the placebo plus paclitaxel group. Median OS was 19.1 months with capivasertib plus paclitaxel and 12.6 months with placebo plus paclitaxel (HR: 0.61). More importantly, in patients with *PIK3CA*/*AKT1*/*PTEN*-altered tumors, median PFS was 9.3 months with capivasertib plus paclitaxel and 3.7 months with placebo plus paclitaxel (HR: 0.30). AKT inhibitors are also active in different stages of clinical trials along with PARP inhibitors. Preclinical studies have demonstrated a synergistic combination of PARP and PI3K-AKT pathway inhibitors in *BRCA1/2*-deficient and -proficient TNBC tumors [50,51,52]. Recently reported trial data of a combination of olaparib plus capivasertib with *BRCA1/2* wild type vs. mutant TNBC patients is also encouraging [53]. Clinical benefit was observed in patients (44.6%) harboring tumors with germline *BRCA1/2* mutations and *BRCA1/2* wild-type cancers with or without somatic DDR and/or the PI3K–AKT pathway alterations. Pharmacodynamic data confirmed pGSK3β suppression, increased pERK, and decreased BRCA1 expression. Combination therapy of AKT inhibitor and Immunotherapy is also active in a clinical trial. A phase III trial is evaluating the triplet combination of atezolizumab (anti-monoclonal antibody of PD-L1) plus ipatasertib and a taxane in locally advanced or metastatic TNBC patients (*NCT 04177108*).

Alpelisib is an oral p110α inhibitor and was FDA approved in 2019 in combination with anti-estrogen for postmenopausal women with HR+/HER2-negative disease, *PIK3CA* mutated advanced or metastatic breast cancer patients [54]. Recently, Novartis has initiated a clinical trial and is recruiting patients for a Phase III, multicenter, randomized, double-blind, placebo-controlled study to assess the efficacy and safety of alpelisib in combination with nab-paclitaxel in patients with advanced TNBC with either *PIK3CA* mutation or PTEN-loss without *PIK3CA* mutation (NCT04251533; Novartis Reference Number: CBYL719H12301).

Androgen receptor pathway: The LAR subtype is characterized by the expression of the androgen receptor (AR) and its downstream effectors. Both AR mRNA and AR protein expression are highly expressed in this subtype of TNBC [11,12]. Meta-analyses led by Qu and Wang, incorporating over 4000 cases of TNBC, demonstrated AR+ status to be associated with better DFS and OS [55,56]. A Translational Breast Cancer Research Consortium phase II study was the first to evaluate anti-androgen therapy in TNBC patients selected by AR status (NCT00468715). The 6-month CBR was 19% for bicalutamide, and the median PFS was 12 weeks [57]. The efficacy of enzalutamide was also evaluated in a phase II study in TNBC patients with locally advanced or metastatic AR-positive tumors. Of 118 patients enrolled, CBR at 16 weeks was 25% in the ITT (intention-to-treat) population and 33% in the evaluable subgroup. Median PFS was 2.9 months in the ITT population and 3.3 months in the evaluable subgroup. Median OS was 12.7 months in the ITT population and 17.6 months in the evaluable subgroup [58]. Both bicalutamide and enzalutamide were well tolerated. Combination therapy with AR inhibitor was evaluated either with a CDK4/6 inhibitor (NCT02605486; NCT03090165) or PI3K inhibitor (NCT 01884285). Preclinical studies demonstrated that the combination therapy with a PI3K inhibitor (GDC-0941 or a dual PI3K/mTOR inhibitor GDC-0980) and AR inhibitor (bicalutamide) has an additive apoptotic effect in AR+ TNBC cell lines [59]. Similarly, a combination of the mTORC1 inhibitor rapamycin and AR inhibitor (enzalutamide) has also shown additive effect in LAR TNBC cell lines and a LAR xenograft model [60].

Angiogenic pathway: The anti-angiogenic therapy in TNBC is encouraged due to highly proliferative and the importance of VEGF in the micro-vascular proliferation of the disease. *TP53* alterations correlate (especially DNA binding domain mutations) with response to VEGF/VEGFR Inhibitors. DNA binding domain mutations have the capacity to upregulate VEGFA and VEGFR2, and VEGF/VEGFR inhibitor treatment was efficacious in combination in *TP53* mutated tumors [61,62]. Basal-like tumors showed a very high frequency of TP53 mutations (~80%) [45]. In the RIBON-1 trial, adding avastin (anti-VEGF antibody) to capecitabine increased PFS from 4.2 to 6.1 months (HR: 0.72) in the TNBC patients [63]. The RIBON-2 trial was initiated to investigate various chemotherapies with or without avastin as a second-line treatment of metastatic breast cancer. In TNBC patients, improvement in PFS with avastin was significant (median PFS 6.0 vs. 2.7 months) compared to chemotherapy alone, and also a trend towards improved OS was observed (HR: 0.62) [64]. Multi-kinase inhibitors such as sunitinib and sorafenib have shown some activity in breast cancer trials with significant in TNBC subgroups, 15% response rate was reported for sunitinib in a phase II trial [65]; however, neither drug is currently approved in TNBC settings.

Wnt-beta-Catenin pathway: The Wnt-beta-catenin pathway is a critical oncologic driver of several epithelial carcinomas, including TNBC tumors. We and others previously reported the importance of upregulation of the Wnt-beta-Catenin pathway in metastatic phenotypes to anti-apoptotic activates in TNBC cells [66,67,68]. TNBC patients with upregulated Wnt signaling often have a poor prognosis [69]. PORCN inhibitors, Wnt ligand antagonists, and FZD antagonists have been examined in several clinical trials. The novel humanized antibody targeting FZD7 (SHH002-hu1) significantly enhanced the anti-TNBC capacity of avastin and showed the potential of preventing TNBC recurrence [70]. Ipafricept (OMP-54F28) is a recombinant-fusion protein-containing combination of the extracellular ligand-binding domain of the human FZD8 receptor and human IgG1 Fc fragment [71,72]. Ipafricept blocks the Wnt-beta-catenin signaling pathway by playing as a decoy receptor while binding and confiscating Wnt ligands. Since it has a capacity to bind all Wnt proteins; hence it functions as a broad spectrum Wnt antagonist [73]. Since its development, Ipafricept has been used in clinical trials in various solid tumors and different chemotherapy regimens. Recently, it has been shown to encourage clinical efficacy in OB-GYN cancer, especially in recurrent platinum-sensitive ovarian cancers, along with paclitaxel and carboplatin. Overall, 28 patients of the ITT had a complete or partial response (CR or PR). CR was reported in 29.7% of patients. Median PFS was 10.3 months, and OS was 33 months (NCT02092363) [73]. The data is highly encouraging to initiate a new clinical trial in TNBC patients, especially in patients with Wnt-beta-catenin pathway upregulation.

Epigenetic pathway: Epigenetic enzyme EZH2 (histone methyltransferase) is a member of the polycomb repressive complex 2 (PRC2) and catalyzes the trimethylation of lysine 27 on histone 3 (H3K27me3) [74]. Phosphorylated EZH2 at threonine 416 (T416) by cyclin E/CDK2 was reported in a high percentage of TNBC patients, which led to poor survival [75]. It has also been reported recently, EZH2 is significantly higher expressed in breast tumors with a *BRCA1* abrogation (mutations as well as BRCA1-promoter methylation). Hence, EZH2 could be used as a predictive biomarker to identify patients according to their likelihood to benefit from intensified DSB-inducing platinum-based chemotherapy independent of BRCA1-like status [76]. Recently EZH2 inhibitor, tazmetostat, has been FDA approved in relapse or refractor follicular lymphoma whose tumors are positive for an EZH2 abrogation. Another epigenetic mechanism-based therapy exploits the lack of ER expression due to hypermethylation of ER promoter. Several epigenetic-based therapies are being studied in the preclinical and clinical trials [77,78]; (NCT01349959). Shira Yomtoubian and colleagues recently demonstrated from their elegant preclinical studies that EZH2-high basal-like 1 and mesenchymal subtypes have exquisite sensitivity to EZH2 inhibition compared with the EZH2-low luminal androgen receptor subtype. EZH2-high basal cells to a luminal-like phenotype by de-repressing GATA3 and renders them sensitive to endocrine therapy. Their study suggests that the benefit of EZH2 inhibition may be derived through transcriptional activation of GATA3 [79]. Future trial data will guide the combined use of epigenetic drugs with PARP inhibitors or with immune therapy.

Transcription pathway: As an RNA polymerase II inhibitor, lurbinectedin inhibits oncogenic transcription. It prevents the binding of the transcription factors to their sequences, and therefore, it inhibits transcription, which is responsible for cell division and eventually leads to cell death or apoptosis. In addition to blocking this in tumor cells, it also inhibits transcription in the tumor-associated macrophages, and therefore it can affect the tumor microenvironment as well. Recently FDA approved the use of lurbinectedin for metastatic small cell lung cancers. Lurbinectedin is active in clinical trials in 9 different tumor types, including metastatic advanced breast cancer with *BRCA1/2* mutated patients. Multicenter phase II study of lurbinectedin in *BRCA* mutated advanced breast cancer showed promising activity. ORR in *BRCA1/2* mutated patients was 41% compared to 9% in BRCA wild-type patients. More interestingly, patients with *BRCA2* mutations showed an ORR of 61%, median PFS of 5.9 months, and median OS of 26.6 months [80].

Cancer being a genetic disease, genomic sequencing is one of the best tools to identify the driver gene alterations, and accordingly, to match the drug(s). Genomic-based molecular interrogation reveals that metastatic cancers, including TNBCs, are vastly complex and individually distinct. Over the past decade, clinical trials have evolved extensively but primarily target one biological variant/one driver gene alteration. Most targeted therapies are designed to inhibit single gene alteration, but co-alterations may affect the efficacy of these therapies. NGS provides us the opportunity to shift the treatment approach from one drug/one gene alteration to a combination treatment approach. The future of clinical trials depends on increasing precise biomarker-based adaptive trials and the N-of-one-like study approach. The integration between bench and bedside will be seamless to ensure constant translational feedback to aid tailor/precision treatment. In today’s world of precision medicine, most of the patient-centric clinical trials were conducted based on single-gene (driver alteration for organ-type cancers) alterations using the targeted drug in combination with standard chemo or immune checkpoint inhibitor. A futuristic approach will need to be developed where multiple driver alterations will be targeted simultaneously by customizing the treatment to block the multiple pathways within tumor cells due to co-mutations of more than one oncogenic pathway. In today’s world of precision medicine, most of the patient-centric clinical trials were conducted based on single-gene (driver alteration for organ-type cancers) alterations using the targeted drug in combination with standard chemo or immune checkpoint inhibitor. A futuristic approach will need to be developed where multiple driver alterations will be targeted simultaneously by customizing the treatment to block the multiple pathways within tumor cells due to co-mutations of more than one oncogenic pathway. Such an approach can be complemented with real-time monitoring of the effect of drug treatment as well as the development of drug-induced clonal resistance identified from the longitudinal liquid-biopsies (CTC and ctDNA).

## 5. Walking Forward

Precision medicine has exploited NGS and also gene/immune system-mediated treatment approaches to transform the outlook for lethal/metastatic cancers, including breast cancers. Genomically-driven molecular interrogation reveals that metastatic cancer, including TNBC, is vastly complex and individually distinct. Hence, the optimized treatment requires combination therapies rather than monotherapy and also patient-centric customized therapy (like the N-of-one treatment approach). It has been established that targeting a larger fraction of identified molecular alterations (depends on the comfort range of the oncologists), yielding a higher “matching score”, was correlated with significantly improved clinical benefit rates, as well as longer progression-free and overall survival rates [81]. A better knowledge of the immunosuppressive role played by the tumor microenvironment has also been important to select a combination of immune checkpoint inhibitors (e.g., a combination of PD-1 or PD-L1 + CTLA4 inhibitors). It may require a dose reduction of specific drugs, the right scheduling of those drugs, and also the constant monitoring of the patient. It has been established that precision dosing (especially for combination therapies) for an individual patient produces desired pharmacological benefits while minimizing drug-related adverse events [82]. It is clear that the future of TNBC therapeutics relies on increasing precise biomarker-based adaptive trials and N-of-one like study approach. For instance, a phase Ib/II, open-label, umbrella study evaluating the efficacy and safety of multiple targeted treatments in patients with metastatic TNBC who had disease progression during or following standard treatment with chemotherapy (FUSCC Refractory TNBC Umbrella trial, NCT03805399). The future holds much promise, and as more information and understanding is acquired, treatment regimens will increasingly incorporate clinically validated biomarker assays, including ctDNA and integration of newer approaches like studying the circulating tumor cells (CTC) that will be of great benefit to these patients. The non-invasive testing of ctDNA and CTC may guide real-time disease monitoring as well as early therapy modifications.

## 6. Limitations

We did not discuss all clinical trials’ details; we also deliberately have not included the drug-induced adverse events and the resistance mechanisms. We also avoided preclinical data, although we admit that preclinical data play a very important for novel therapeutic opportunities. Likewise, we did not review the transcriptomics and proteomics analysis which are component clinical strategies as transcriptomics/proteomics-mediated clinical trials are not routinely carried out yet.

## 7. Conclusions

TNBC is an aggressive subtype of breast cancers with intrinsic molecular and immunological heterogeneity. Recently, several molecularly targeted agents have been developed and approved for the treatment of TNBC patients. Several combination therapies are active in the various stages of clinical trials, and these combinations have the potential to transform the therapeutic landscape of TNBC patients in the near future. The important clinical question is how to sequence immunotherapy, targeted therapies, and standard chemotherapy in TNBC patients with germline BRCA1/2 alteration or PI3K-AKT pathway upregulation with PD-L1 positive tumors. For instance, in PTEN-mutated patients, AKT inhibitor may be used upfront, followed by immunotherapy, since reports suggest that loss of PTEN may contribute to immunotherapy resistance [83,84]. Another approach would be simultaneous administration of immunotherapy and targeted therapy drugs. Despite the major development/approval of new drugs in TNBC patients’ care, drug resistance will eventually evolve. The future holds the knowledge of tumor-stromal interactions underlying the development of drug resistance as well as immune evasion. Riding on it a newer combination(s) with more potent/targeted drugs and innovative drug administration strategies will provide a better outcome from clinical trials. Tumor genotyping, along with ctDNA profiling and also identifying germline mutation, may help to identify actionable mutations and lead to a mutation-specific treatment or patient enrollment in suitable clinical trials. However, their prognosis values need further broad-scale evaluation. Challenge remains to change the treatment paradigm/clinical trial setting from a one targeted drug/one genetic alteration approach to a combination treatment strategy. Another hurdle is the complexity of the tumor-immune microenvironment (TME) and its real-time evolution in response to drug treatment. Such an ongoing alteration in the TME and its cross-talk with the tumor compartment suggest that a single biomarker-based (e.g., PD-L1 expression status or TMB status) strategy cannot select patient who should receive or not receive immune checkpoint inhibitor.

## Figures and Tables

**Table 1:** Promising treatment options with drugs that need FDA approval.

Name of the Drug(s)	Target	Patients Types	Clinical Trial Number
Ladiratuzumab vedotin (LV)	Zinc transporter LIV1 conjugated with MMAE	Advanced or metastatic TNBC	NCT01969643
LV plus pembrolizumab	LIV1 and PD-1	Front line therapy in TNBC patient	NCT03310957
NBE-002	ROR1 conjugated with anthracycline	Advanced solid tumors, including TNBC	NCT044410099
Trastuzumab deruxtecan	HER2 conjugated with topoisomerase1 inhibitor	HER2-low un-resectable and/or metastatic breast cancer	NCT03734029
Ipataserib (GDC-0068) (LOTUS trial)	AKT	PI3K-AKT activated TNBC	NCT02162719
Ipataserib (GDC-0068) plus atezolizumab	AKT and PD-L1	Locally advanced unresectable or metastatic triple-negative breast cancer	NCT04177108
Capivasertib (AZD5363) (PAKT trial)	AKT	Advanced or metastatic TNBC	NCT02423603
Alpelisib	P110 alpha (catalytic subunit of PI3K)	Advanced TNBC	NCT04251533
Bicalutamide	Androgen receptor	Metastatic TNBC	NCT00468715
Enzalutamide	Androgen receptor	Advanced, androgen receptor-positive, TNBC	NCT01889238
Bicalutamide plus palbociclib	Androgen receptor and CDK4/6	AR(+) metastatic breast cancer, including TNBC	NCT02605486
Bicalutamide plus ribociclib	Androgen receptor and CDK4/6	AR(+) TNBC	NCT03090165
AZD8186 (single agent) and AZD8186 plus abiraterone acetate	PI3K beta/delta and androgen receptor	TNBC	NCT01884285
Avastin plus everolimus	VEGF and mTORC1	Locally advanced TNBC with tumors predicted insensitive to standard chemotherapy	NCT02456857
Avastin	VEGF	Metastatic TNBC	NCT03577743
Avastin plus atezolizumab	VEGF and PD-L1	Metastatic TNBC	NCT04739670
Azacitidine plus entinostat	DNA methyltransferase and HDAC	Advanced breast cancers including TNBC	NCT01349959
Lurbinectedin	RNA polymerase II	Metastatic breast cancer, pancreatic cancer, and metastatic colorectal cancer	NCT02210364

## Data Availability

Not applicable.

## Abbreviations

ADC	antibody–drug conjugate
AR	androgen receptor
CBR	clinical benefit rate
CR	complete response
ctDNA	circulating tumor DNA
CPS	combines positive score
CTC	circulating tumor cells
CTLA4	cytotoxic T-lymphocyte-associated protein 4
DCR	disease control rate
DFS	disease-free survival
ER	estrogen receptor
HR	homologous recombination
HR	hazard ratio
HER2	epidermal growth factor receptor 2
ITT	intention to treat
IL	interleukin
NGS	next-generation sequencing
ORR	objective response rate
OS	overall survival
PARP	poly (ADP ribose) polymerase
PR	progesterone receptor
PR	partial response
PD-1	programmed cell death protein 1
PD-L1/2	programmed death-ligand 1/2
PFS	progression-free survival
TNBC	triple-negative breast cancer
VEGF	vascular endothelial growth factor
